# Comparing Mixed Reality and Two-Dimensional Imaging in Mandibular Fracture Classification: A Prospective Randomized Study in Medical and Dental Students

**DOI:** 10.3390/jcm15083018

**Published:** 2026-04-15

**Authors:** Valerian Dirr, Leyla Halter, Maximilian Ries, Gregoire Longchamp, Raphael Ferrari, Harald Essig, Maximilian E. H. Wagner

**Affiliations:** Department of Cranio-Maxillo-Facial and Oral Surgery, University Hospital Zurich, Rämistrasse 100, 8091 Zurich, Switzerland; leyla.halter@usz.ch (L.H.); maximilian.ries@usz.ch (M.R.); gregoire.longchamp@usz.ch (G.L.);

**Keywords:** mixed reality, virtual reality, medical education, dental education, mandibular fractures

## Abstract

**Background:** Oral and cranio-maxillofacial (OCMF) surgery is a complex specialty that requires detailed anatomical knowledge and, in fracture care, the ability to interpret imaging accurately. Mixed reality (MR) may improve spatial understanding in anatomy-based disciplines, but its value for teaching mandibular fracture classification remains uncertain. **Methods**: Medical and dental students at the University of Zurich were randomized 1:1 to classify four unilateral mandibular fractures using either MR or conventional two-dimensional (2D) imaging. Primary outcomes were perceived usefulness, ease of use, learning, and user satisfaction, assessed with a 15-item usability questionnaire. Secondary outcomes were fracture-classification accuracy and time to fracture classification. **Results:** Forty medical and dental students were included. Baseline characteristics were comparable between groups, and overall fracture-classification accuracy did not differ significantly between MR and 2D. Both groups became faster across successive cases, indicating a learning effect, although the 2D group completed classifications more quickly overall. MR participants reported higher scores for learning and user satisfaction, whereas the 2D group rated ease of use more favorably. **Conclusions:** MR increased user satisfaction but did not improve fracture-classification accuracy compared with 2D imaging. When integrated thoughtfully into OCMF education, MR may complement, rather than replace, conventional imaging approaches.

## 1. Introduction

Oral and cranio-maxillofacial (OCMF) surgery is a complex surgical specialty that requires several years of postgraduate training and, in many European countries, degrees in both medicine and dentistry. Because of the close proximity of critical anatomical structures in the head and neck, the specialty demands a thorough understanding of three-dimensional anatomy. Consequently, innovative learning methods such as Virtual Reality (VR), Augmented Reality (AR), and MR may be particularly valuable.

VR generates an entirely immersive virtual environment with computer-generated graphics and sounds by using head-mounted devices (HMDs), motion sensors and controllers equipped with haptic feedback [1]. AR overlays digital images with the real environment, creating an immersive space for the user. MR uses aspects of both VR and AR by using HMDs, such as the Microsoft HoloLens2, to combine virtual and real elements. Computer graphics are integrated with real-world elements, allowing users to interact with virtual and physical elements simultaneously and therefore creating an interactive learning environment. MR technologies have advanced substantially in recent years and are increasingly being explored as learning and teaching tools in higher education, particularly in medicine, as they allow repeated practice and may increase student motivation and engagement [2,3,4].

Recent research has examined these technologies in OCMF surgery for surgical training, intraoperative navigation, and patient-specific preoperative planning [5,6,7,8,9]. At present, they are used mainly in implantology and orthognathic surgery for preoperative planning and intraoperative guidance [10,11,12,13]. In education, VR and AR have shown value in preclinical training and surgical-skill development [14,15,16,17].

MR technologies have advanced substantially in recent years and are increasingly being explored as learning and teaching tools in higher education, particularly in medicine [3]. Head-mounted devices (HMDs), such as the Microsoft HoloLens 2, combine virtual and real elements to create interactive learning environments. These systems allow repeated practice and may increase student motivation and engagement [4].

Mandibular fractures are a common and clinically relevant problem in OCMF practice [18]. The AO Foundation OCMF classification system provides a standardized method for categorizing fractures according to severity and treatment complexity across four hierarchical levels [19]. Given the anatomical complexity of the craniofacial region, novel educational strategies are needed to promote sustainable, competency-based learning. Although MR has shown potential to enhance spatial understanding and clinical skill acquisition, its role in undergraduate surgical education, particularly within OCMF, remains insufficiently studied [20,21,22,23,24,25,26]. Therefore, we evaluated the impact of MR on perceived usefulness, user satisfaction, ease of use, classification accuracy, and time to classification compared with conventional 2D imaging.

## 2. Materials and Methods

### 2.1. Study Participants

The study included third- to sixth-year medical and dental students from the University of Zurich. Participants were randomized 1:1 to use either 2D imaging or MR technology. Each participant analyzed four cases of single unilateral mandibular fractures. The patient cases were pseudo-anonymized and assigned numbers from 1 to 11. For each case, both a 2D image dataset and a 3D MR dataset were prepared. Four cases were randomly assigned to each participant using an online randomization tool. After completing all four cases, participants completed a web-based questionnaire capturing demographic data, AO CMF mandible classification responses, fracture-classification time, and the usability questionnaire.

Cases meeting the inclusion criteria were treated at the University Hospital Zurich between 1 January 2020 and 31 December 2024 (see Appendix A for the inclusion and exclusion criteria). Data collection was performed at the University of Zurich and the University Hospital Zurich. In total, 11 patient cases with mandibular fractures were selected from the authors’ institution and independently evaluated by one junior and one senior surgeon.

### 2.2. Outcome Variables

The primary outcome was the perceived usefulness, ease of use, and user satisfaction of MR compared with conventional 2D imaging. Usability was assessed with the Computer System Usability Questionnaire, Version 3. This instrument is derived from the Computer System Usability Questionnaire (CSUQ) developed by Lewis in 1995 and is used to assess perceived satisfaction, system usefulness, information quality, and interface quality for software applications and other computer systems. The CSUQ is a revised version of the Post-Study System Usability Questionnaire (PSSUQ). Both instruments measure overall satisfaction, but the CSUQ uses present-tense wording and is therefore well suited to non-laboratory field evaluations.

The questionnaire used in this study consisted of 15 items rated on a 7-point Likert scale ranging from 1 (strongly agree) to 7 (strongly disagree) (see Appendix A for the questionnaire items) [27].

**Figure A1:**
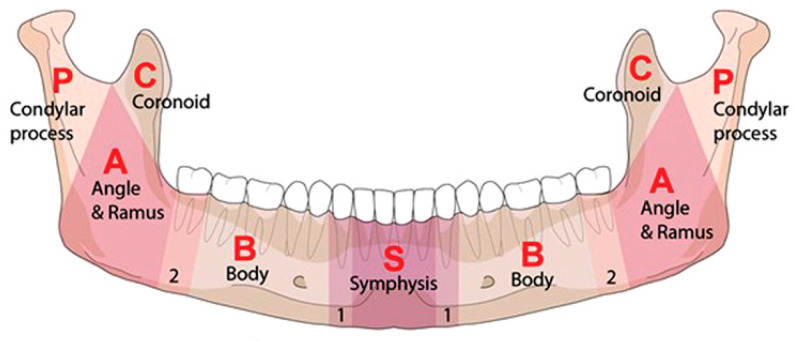
The AO CMF mandible Level I classification. Panoramic view of the mandible with a full permanent dentition, as presented to the participants prior to the examination. The mandible is divided into nine topographical regions with the symphysis (S) in the anterior central position. All other regions, including the body (B), angle/ramus (A), coronoid (C), and condylar process (P), are symmetric. Two pairs of transitional zones are assembled between the S and B (1  =  anterior transition zone) and between the B and A (2  =  posterior transition zone) [43].

Secondary outcomes were classification accuracy and time to fracture classification. Fractures were classified using the AO CMF Level 3 classification system, including side and anatomical location, displacement, displacement direction, and fragmentation [19]. Classification time was measured in seconds from the moment participants first saw the images until they were ready to record their final classification. Before the assessment, participants received a five-minute introduction and reviewed an explanatory image of the classification system (Figure A1) adapted from Mittermiller et al. [28]. Participants with prior knowledge of the classification system were excluded.

### 2.3. Institutional Review and Ethics

According to the Cantonal Ethics Commission of Zurich, formal ethical approval was not required for this study design (Req-2024-00889).

### 2.4. Augmedit Lumi Software and DeepUnity Review

**Figure 1:**
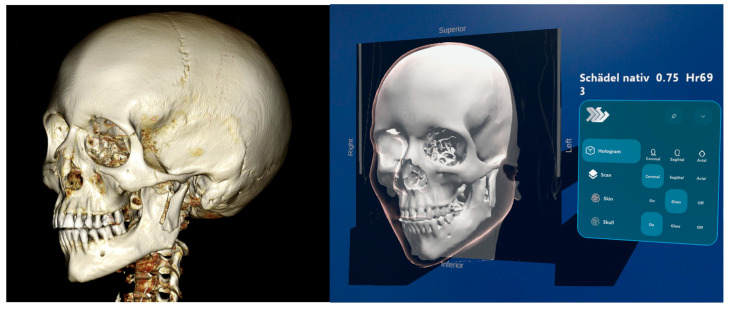
(**Left**) Screenshot of the 3D reconstruction of a condylar process fracture, as demonstrated to the students in DeepUnity Review on a hospital monitor. (**Right**) View through the HoloLens in the Augmedit Lumi software used to visualize data set. The hologram was intentionally positioned against a neutral background to improve visibility. It is controlled by finger gestures detected by the headset’s sensors. Various settings allow different modes of visualization. In this example, the osseous reconstruction, coronal section, and soft-tissue structures displayed in transparent mode are activated.

CT images were used to create volumetric models for educational purposes. The CT datasets were retrieved from the institutional Picture Archiving and Communication System (PACS; AGFA Healthcare, Dübendorf, Switzerland) via the hospital information system KISIM (Cistec, Zurich, Switzerland) at the University Hospital Zurich. The DICOM files were processed with Lumi software (LumiNe Lite, Augmedit, Amsterdam, The Netherlands), which converts standard DICOM data into interactive 3D volume renderings suitable for visualization with HMDs. Microsoft HoloLens 2 was used to display the holographic models in MR. To ensure compliance with data-protection regulations, a one-way encryption algorithm generated a unique anonymized identifier for each patient (Figure 1).

Participants in the second group used DeepUnity Review (Dedalus Healthcare Systems Group), a non-diagnostic viewer for DICOM and non-DICOM medical images. The platform is designed for clinical image review and multidisciplinary collaboration and integrates with hospital and radiology information systems (Figure 1).

### 2.5. Statistical Analysis

Descriptive statistics were used to summarize the data. Depending on the distribution, continuous variables are reported as means ± standard deviations (SDs) or medians with interquartile ranges (IQRs). Categorical variables are reported as counts and percentages. Continuous variables were compared using the *t*-test or Mann–Whitney U test, and categorical variables were compared using the chi-square test or Fisher’s exact test. To assess within-group changes over time, the Friedman test was used for repeated measurements. No imputation was performed for missing data. No formal a priori sample size calculation was performed, as this was an exploratory study without prior data to inform effect size estimates. Statistical significance was defined as a two-sided *p*-value < 0.05. Interrater reliability was assessed using overall percent agreement and Krippendorff’s alpha for nominal data to evaluate the degree of agreement among students within each imaging modality, as it accommodates varying numbers of raters per subject. Krippendorff’s alpha values were interpreted as follows: α < 0.00 = poor; 0.00–0.20 = slight; 0.21–0.40 = fair; 0.41–0.60 = moderate; 0.61–0.80 = substantial; 0.81–1.00 = almost perfect agreement. All analyses were performed with Stata 16.1 (StataCorp, College Station, TX, USA).

### 2.6. Generative Artificial Intelligence Statement

During the preparation of this manuscript, the authors used OpenAI tools exclusively for spelling and grammar correction. The authors reviewed and approved the final manuscript and take full responsibility for the content of this publication.

## 3. Results

### 3.1. Baseline Characteristics

A total of 40 students were randomized equally to the MR group (*n* = 20) and the 2D group (*n* = 20). Baseline characteristics were generally balanced, with no significant differences in age (median 23 vs. 22 years, *p* = 0.112), sex distribution (45% vs. 55% female, *p* = 0.527), field of study (medicine vs. dentistry, *p* = 1.000), academic year (*p* = 0.487), or symptoms (*p* = 0.072). Image-quality ratings were comparable, with most datasets rated as good or very good in both groups (*p* = 0.191) (Table 1).

### 3.2. Time to Detect Fractures

The time required to identify mandibular fractures decreased progressively across cases in both groups, indicating a learning effect (Table A1). In the MR group, the median detection time decreased from 377.5 s in the first case to 186.5 s in the fourth case. Although detection times were initially longer in the MR group than in the 2D group, this difference narrowed over time and reached statistical significance only in the fourth case (186.5 vs. 114 s, *p* = 0.011). Missing data were minimal (<3% per case) and were evenly distributed between groups.

**Figure 2:**
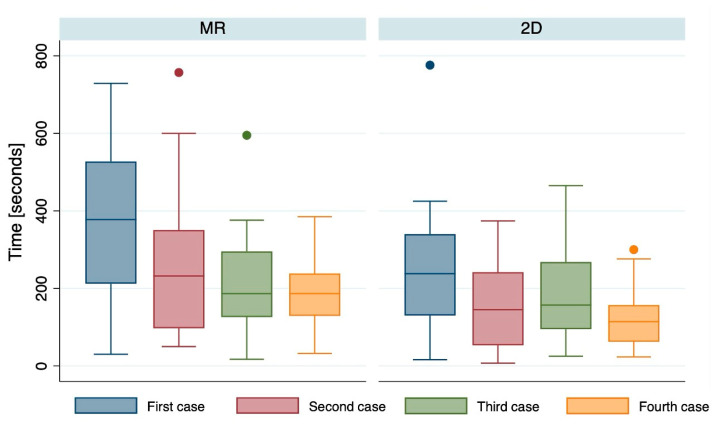
Time to detect the fracture by case. A Friedman test was conducted to compare repeated measures of time within each group and yielded statistically significant differences over the four cases in both the MR group and the 2D group. Missing data: first case = 1 (2.5%); second case = 1 (2.5%); third case = 1 (2.5%); fourth case = 1 (2.5%).

**Figure A2:**
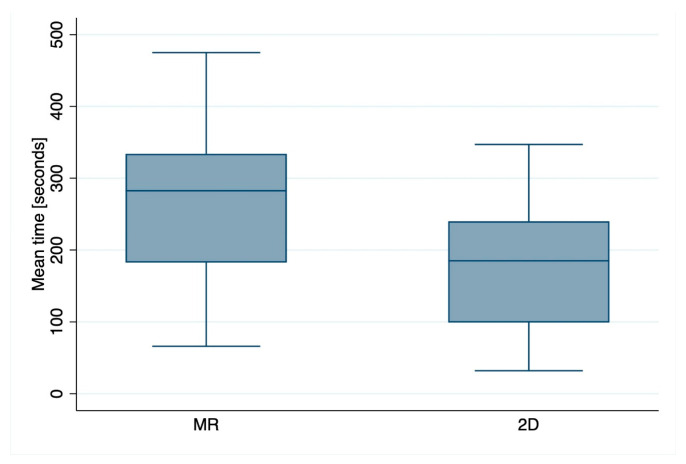
Mean time in seconds to detect the fracture. The mean time for fracture identification was 259.7 s (SD 108.7 s) in the MR group compared with 176.9 s (SD 91.5 s) in the 2D group. Missing data: MR group, 4 cases (2.5%).

Both groups showed a significant reduction in detection time across cases (*p* = 0.023 for MR; *p* = 0.0002 for 2D) (Figure 2). When averaged across all cases, the mean detection time was significantly longer in the MR group than in the 2D group (259.7 ± 108.7 s vs. 176.9 ± 91.5 s, *p* = 0.024) (Figure A2).

### 3.3. Accuracy in Fracture Classification & Interrater Reliability

Accuracy in identifying specific fracture characteristics improved over time in both groups (Table A2). Determination of fracture side was consistently high across cases, with no significant between-group differences (all *p* > 0.48). Localization accuracy was initially lower in the MR group (20.0% vs. 50.0%, *p* = 0.047) but improved in later cases and was comparable with the 2D group by the fourth case (36.8% vs. 47.4%, *p* = 0.511). Similar patterns were observed for fragmentation and displacement. Displacement direction remained challenging for both groups, with no significant between-group differences.

Overall accuracy across the four cases was comparable between MR and 2D (Table A4). Sixty percent of participants in the MR group achieved accurate classifications in all cases compared with 40% in the 2D group (*p* = 0.432). Likewise, the proportions achieving very accurate (75% vs. 60%, *p* = 0.780) and extremely accurate (80% vs. 65%, *p* = 0.764) classifications were numerically higher in the MR group, although these differences were not statistically significant.

Per-case accuracy also improved progressively in both groups (Table A3). In the first case, accurate classifications were less frequent in the MR group than in the 2D group (20.0% vs. 45.0%, *p* = 0.091). However, MR participants improved over time and reached comparable performance by the fourth case (36.8% vs. 42.1%, *p* = 0.740). Similar patterns were observed for very accurate and extremely accurate classifications, with no statistically significant differences across cases. These findings suggest a learning effect in both groups, with the MR group narrowing the initial performance gap.

Interrater reliability for fracture side classification was moderate in the MR group (Krippendorff’s α = 0.54) and fair in the 2D group (α = 0.37). For fracture localization, agreement was fair in the MR group (α = 0.21) and moderate in the 2D group (α = 0.56).

### 3.4. Usability Questionnaire

**Figure 3:**
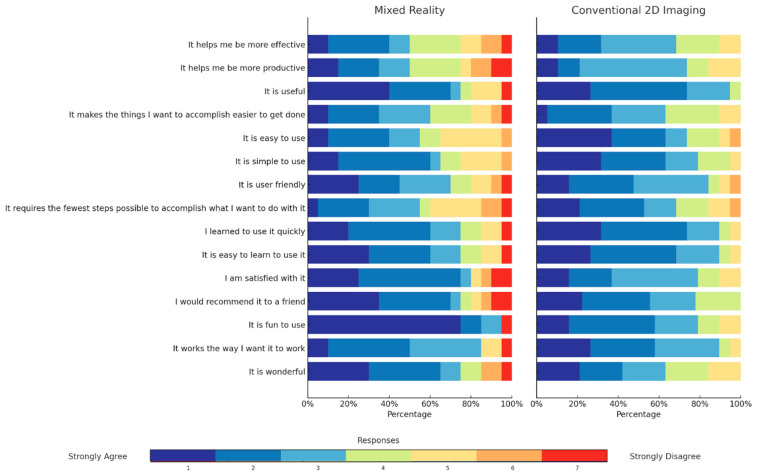
A stacked horizontal Likert plot showing the distribution of questionnaire responses by item and study group.

Perceived usefulness, ease of use, learning, and satisfaction were evaluated using the usability questionnaire (Table 2 and Figure 3). Overall usefulness scores did not differ significantly between groups (*p* = 0.628). Because lower scores indicate more favorable ratings, the 2D group rated ease of use significantly more favorably than the MR group (median 10 vs. 12.5, *p* = 0.003). This difference was driven primarily by items related to intuitive handling and workflow integration (items 5 and 8, both *p* < 0.001).

MR participants reported greater perceived learning benefits for item 9 (*p* = 0.019), and the overall learning score also tended to favor MR, although the difference did not reach statistical significance (*p* = 0.054). Satisfaction was generally high in both groups; however, MR participants rated several aspects more favorably, with lower scores for enjoyment and frustration (items 11, 13, and 14; *p* = 0.006, *p* < 0.001, and *p* = 0.049, respectively). Overall satisfaction scores were significantly better in the MR group (median 9 vs. 12, *p* = 0.013), whereas total usability scores did not differ significantly (*p* = 0.443).

As shown in Figure 3, MR participants rated several items related to engagement and satisfaction more favorably, whereas the 2D group tended to rate ease of use more favorably. These findings are consistent with the significant between-group differences in overall ease-of-use and satisfaction scores.

## 4. Discussion

In this prospective randomized study, overall usability was comparable between the two groups. However, user satisfaction was significantly higher in the MR group, whereas the 2D group showed significantly greater ease of use and shorter time to fracture identification. Only three participants (7.5%) experienced MR-related symptoms during the study. This study addresses a relatively unexplored area by evaluating learning and training outcomes associated with MR and conventional 2D imaging in mandibular fracture classification among undergraduate medical and dental students [29,30].

The findings suggest that MR may add educational value in a demanding clinical setting. Greater user satisfaction with MR may support long-term learning by increasing student engagement and motivation. We believe this is attributable to students having the opportunity to engage with this new technology. It may also foster openness to innovative learning methods within a supportive educational environment. These observations are consistent with previous work highlighting user satisfaction as an important contributor to educational quality and learning effectiveness in medical education [31,32].

The more favorable ease-of-use ratings in the 2D group are plausible because students interact with conventional screens in their daily academic work. Before the first timed case, neither group received detailed instructions on use of the respective software solutions, and participants were expected to familiarize themselves with the systems independently. Both groups nevertheless showed a comparable reduction in completion time across cases. Because MR is not yet fully established in contemporary medical education, repeated practice may further reduce the absolute time difference between groups. A recent study in Scientific Reports showed that prolonged exposure to MR can improve task performance as users adapt to the interface [33].

Notably, MR did not improve fracture-classification accuracy compared with the conventional 2D approach. One explanation may be the generally modest accuracy observed in both groups. The task appears to have been challenging when performed under time pressure and without prior preparation or clinical experience, which likely limits correct classification even at lower classification levels. This study addresses a gap in OCMF education by evaluating MR for mandibular fracture classification. Studies in other surgical domains suggest that MR can improve fracture identification and spatial understanding, particularly among more experienced users [34,35]. Conversely, work in neurosurgical education has shown the benefits of immersive 3D learning more broadly, while the optimal role of VR versus MR remains under discussion [36]. Interrater reliability analysis indicate that agreement was generally fair to moderate, which is consistent with the complexity of the AO CMF classification system and the limited prior experience of the undergraduate student participants. The higher localization agreement in the 2D group may reflect students’ greater familiarity with conventional imaging.

The present findings support the integration of MR technologies into medical and dental education as part of a broader shift toward competency-based learning, in which repeated, self-directed practice is essential for skill acquisition and clinical proficiency. Unlike traditional lecture-based methods, MR enables immersive, experiential learning that may deepen understanding of complex anatomical relationships such as those encountered in OCMF surgery [37,38]. For long-term educational impact, MR should be integrated into curricula in a structured way, with progressive complexity, feedback, and clinical context. This position is reinforced by the existing literature, which emphasizes that MR yields its greatest educational value when implemented systematically within formal teaching programs. Under these conditions, MR can improve spatial reasoning, procedural confidence, and learner engagement [39,40]. In addition, MR may help address the growing demand for scalable, remote, and asynchronous learning formats in medical education. Cloud-based platforms and head-mounted displays can extend access to high-fidelity simulations beyond the hospital environment, as illustrated in the present study [4,41]. Although the investment required for MR hardware and software remains substantial, the use of MR technology is expanding across multiple fields and may soon become standard in medical education [29,42].

This study has several limitations. First, participants had no prior experience with MR or the Lumi software and received only a brief introduction before starting the task. This limited familiarity may have affected performance, particularly task duration and perceived ease of use. Second, the study evaluated only short-term outcomes after a single session and therefore does not capture the potential effects of repeated MR exposure on long-term retention, transfer of learning, or skill progression. Third, the cohort included only 40 undergraduate students from a single institution, which limits generalizability. In addition, mandibular fracture classification with the AO CMF system is a cognitively demanding task for learners with limited clinical exposure. The immersive nature of MR may also increase cognitive load in novice users, potentially offsetting some of the advantages of enhanced spatial visualization. Finally, the cost of MR systems may limit broader implementation. Future studies should be designed as prospective, multi-institutional, randomized trials including undergraduate students, residents, and consultants with varying levels of surgical experience with clearly defined tasks of increasing complexity to evaluate the benefit of MR in surgical education.

## 5. Conclusions

This study suggests that MR may be a useful adjunct to conventional imaging in medical and dental education, particularly by increasing learner engagement in complex clinical scenarios such as mandibular fracture classification. Although MR did not improve accuracy compared with 2D imaging, it significantly increased user satisfaction and both groups showed a comparable reduction in classification time over successive cases. The possibility of delivering MR-based training outside the hospital through cloud-based platforms also highlights its potential for flexible and scalable education. Broader implementation will require appropriate scaffolding, user training, curricular integration, and careful consideration of cost.

## Figures and Tables

**Table 1:** Baseline characteristics.

	Overall (*n* = 40)	MR (*n* = 20)	2D (*n* = 20)	*p*-Value
Age, years	22.5 (22–24)	23 (22–24.5)	22 (22–23)	0.112 ^1^
Gender, female	20 (50)	9 (45)	11 (55)	0.527 ^2^
Study				1.000 ^2^
Medicine	35 (87.5)	17 (85.0)	18 (90.0)	
Dentistry	5 (12.5)	3 (15.0)	2 (10.0)	
Academic year				0.487 ^2^
Third	7 (17.5)	4 (20.0)	7 (17.5)	
Fourth	31 (77.5)	14 (70.0)	31 (77.5)	
Fifth	1 (2.5)	1 (5.0)	1 (2.5)	
Sixth	1 (2.5)	1 (5.0)	1 (2.5)	

Continuous variables are median (IQR) and discrete variables are *n* (%). ^1^ Mann–Whitney test. ^2^ Fisher’s exact test.

**Table 2:** Usability questionnaire results.

		MR	2D	*p*-Value
Usefulness	Item 1	3.5 (2–4.5)	3 (2–4)	0.182
	Item 2	3.5 (2–4.5)	3 (3–4)	0.233
	Item 3	2 (1–3.5)	2 (1–3)	0.881
	Item 4	3 (2–4)	3 (2–4)	0.540
	Overall usefulness	10.5 (8–17)	12 (7–13)	0.628
Ease of use	Item 5	3 (2–5)	2 (1–4)	<0.001
	Item 6	2 (2–4.5)	2 (1–3)	0.020
	Item 7	3 (1.5–4)	3 (2–3)	0.579
	Item 8	3 (2–5)	2 (2–4)	<0.001
	Overall ease of use	12.5 (8–15.5)	10 (6–14)	0.003
Learning	Item 9	2 (2–3.5)	2 (1–3)	0.019
	Item 10	2 (1–3.5)	2 (1–3)	0.376
	Overall learning	4 (3–7)	4 (3–6)	0.054
Satisfaction	Item 11	2 (1.5–2.5)	3 (2–3)	0.006
	Item 12	2 (1–3.5)	2 (2–3)	0.298
	Item 13	1 (1–1.5)	2 (2–3)	<0.001
	Item 14	2.5 (2–3)	2 (1–3)	0.049
	Item 15	2 (1–3.5)	3 (2–4)	0.072
	Overall satisfaction	9 (7.5–13.5)	12 (9–16)	0.013
Overall score		38.5 (28–47)	34 (30–46)	0.443

## Data Availability

Data deposition in a repository was not possible. Data of the study is available upon request for interested researchers.

## Abbreviations

The following abbreviations are used in this manuscript: OCMF, oral and cranio-maxillofacial; VR, virtual reality; AR, augmented reality; MR, mixed reality; 2D, two-dimensional; HMD, head-mounted device; CT, computed tomography; DICOM, Digital Imaging and Communications in Medicine; PACS, Picture Archiving and Communication System; IQR, interquartile range; SD, standard deviation; CSUQ, Computer System Usability Questionnaire; PSSUQ, Post-Study System Usability Questionnaire; CBCT, cone-beam computed tomography; MRI, magnetic resonance imaging.

## Appendix A

### Appendix A.1. Inclusion Criteria

- Patients > 18 years old.
- Patients presenting with traumatic CMF fractures (mandible, midface, skull base & cranial vault) who received a CT, and/or MRI and/or a CBCT.
- Patients undergoing surgical treatment due to the fractures (mandible, midface, skull base & cranial vault).
- The investigator identifying potential usefulness for 3D assessment of the patient.
- The patient provided general consent for clinical research.

### Appendix A.2. Exclusion Criteria

- Patients < 18 years of age.
- Patients who did not provide general consent for clinical research.

### Appendix A.3. Usability Questionnaire Items

- It helps me be more effective
- It helps me be more productive
- It is useful
- It makes the things I want to accomplish easier to get done
- It is easy to use
- It is simple to use
- It is user friendly
- It requires the fewest steps possible to accomplish what I want to do with it
- I learned to use it quickly
- It is easy to learn to use it
- I am satisfied with it
- I would recommend it to a friend
- It is fun to use
- It works in the way I want to work
- It is wonderful

## Appendix B

**Table A1:** Time to detect the fracture, in seconds.

	MR	2D	*p*-Value
First case	377.5 (212–527.5)	238 (130–340)	0.058
Second case	232 (97–350.5)	145 (53–242)	0.095
Third case	186.5 (126–295.5)	157 (95–268)	0.325
Fourth case	186.5 (129–238.5)	114 (62–159)	0.011

Variables are expressed as median (IQR). Missing data: first case = 1 (2.5%); second case = 1 (2.5%); third case = 1 (2.5%); fourth case = 1 (2.5%).

**Table A2:** Correct identification of fracture characteristics.

	First Case			Second Case			Third Case			Fourth Case		
	MR	2D	*p*-Value	MR	2D	*p*-Value	MR	2D	*p*-Value	MR	2D	*p*-Value
Side	16 (80.0)	16 (80.0)	1.000 *	17 (89.5)	18 (94.7)	1.000 *	16 (80.0)	13 (72.2)	0.709	12 (63.2)	14 (73.7)	0.485
Localization	4 (20.0)	10 (50.0)	0.047	9 (47.4)	10 (52.6)	0.746	6 (30.0)	6 (33.3)	0.825	7 (36.8)	9 (47.4)	0.511
Fragmentation	12 (60.0)	12 (60.0)	1.000	13 (68.4)	9 (47.4)	0.189	13 (65.0)	15 (83.3)	0.200	13 (68.4)	11(57.89)	0.501
Displacement	10 (50.0)	10 (50.0)	1.000	10 (52.6)	14 (73.7)	0.179	6 (30.0)	12 (66.7)	0.024	10(52.63)	11 (57.9)	0.744
Displacement direction	8 (40.0)	7 (35.0)	0.744	7 (36.8)	11 (57.9)	0.194	5 (25.0)	9 (50.0)	0.111	8 (42.1)	8 (42.1)	1.000

Variables are expressed as *n* (%). * Fisher’s exact test.

**Table A3:** Accuracy per case.

	First Case			Second Case			Third Case			Fourth Case		
	MR	2D	*p*-Value	MR	2D	*p*-Value	MR	2D	*p*-Value	MR	2D	*p*-Value
Accurate	4 (20.0)	9 (45.0)	0.091	9 (47.4)	10 (52.6)	0.746	6 (30.0)	5 (27.8)	0.880	7 (36.8)	8 (42.1)	0.740
Very accurate	0	2 (10.0)	0.487 *	4 (21.1)	3 (15.8)	1.000 *	1 (5.0)	3 (16.7)	0.328 *	0	2 (10.5)	0.146 *
Extremely accurate	0	2 (10.0)	0.147 *	3 (15.8)	3 (15.7)	1.000 *	1 (5.0)	3 (16.7)	0.328 *	0	1 (5.3)	1.000 *

Variables are expressed as *n* (%). * Fisher’s exact test.

**Table A4:** Overall accuracy across the four cases.

	MR	2D	*p*-Value
Accurate			0.432
0	12 (60.0)	8 (40.0)	
25%	3 (15.0)	5 (25.0)	
33%	2 (10.0)	1 (5.0)	
50%	1 (5.0)	2 (10.0)	
66%	0	3 (15.0)	
75%	2 (10.0)	1 (5.0)	
Very accurate			0.780
0%	15 (75.0)	12 (60.0)	
25%	4 (20.0)	5 (25.0)	
33%	1 (5.0)	1 (5.0)	
50%	0	1 (5.0)	
66%	0	1 (5.0)	
Extremely accurate			0.764
0%	16 (80.0)	13 (65.0)	
25%	3 (15.0)	4 (20.0)	
33%	1 (5.0)	1 (5.0)	
50%	0	1 (5.0)	
66%	0	1 (5.0)	

Variables are expressed as *n* (%).
